# Managing patients with prediabetes and type 2 diabetes after coronary events: individual tailoring needed - a cross-sectional study

**DOI:** 10.1186/s12872-018-0896-z

**Published:** 2018-08-03

**Authors:** John Munkhaugen, Jøran Hjelmesæth, Jan Erik Otterstad, Ragnhild Helseth, Stina Therese Sollid, Erik Gjertsen, Lars Gullestad, Joep Perk, Torbjørn Moum, Einar Husebye, Toril Dammen

**Affiliations:** 1 Department of Medicine, Drammen Hospital, Vestre Viken Health Trust, Dronninggata 41, 3004 Drammen, Norway; 2 Morbid Obesity Centre, Vestfold Hospital Trust, Tønsberg, Norway; 3 Department of Endocrinology, Morbid Obesity and Preventive Medicine, Institute of Clinical Medicine, University of Oslo, Oslo, Norway; 4 Department of Medicine, Vestfold Hospital Trust, Tønsberg, Norway; 5 Centre for Clinical Heart Research, Department of Cardiology, Oslo University Hospital Ullevål, Oslo, Norway; 6 Faculty of Medicine, University of Oslo, Oslo, Norway; 7 Department of Medicine, Drammen Hospital Trust, Drammen, Norway; 8 Department of Cardiology, Oslo University Hospital Rikshospitalet, Oslo, Norway; 9 Linneus University, Kalmar, Sweden; 10 Department of Behavioural Sciences in Medicine and Faculty of Medicine, University of Oslo, Oslo, Norway

**Keywords:** Secondary prevention, Coronary heart disease, Type 2 diabetes, Prediabetes, HbA_1c_, Risk factor control, Glycaemic control, Psychosocial factors

## Abstract

**Background:**

Understanding the determinants associated with prediabetes and type 2 diabetes in coronary patients may help to individualize treatment and modelling interventions. We sought to identify sociodemographic, medical and psychosocial factors associated with normal blood glucose (HbA1c < 5.7%), prediabetes (HbA1c 5.7–6.4%), and type 2 diabetes.

**Methods:**

A cross-sectional explorative study applied regression analyses to investigate the factors associated with glycaemic status and control (HbA_1c_ level) in 1083 patients with myocardial infarction and/or a coronary revascularization procedure. Data were collected from hospital records at the index event and from a self-report questionnaire and clinical examination with blood samples at 2–36 months follow-up.

**Results:**

In all, 23% had type 2 diabetes, 44% had prediabetes, and 33% had normal blood glucose at follow-up. In adjusted analyses, type 2 diabetes was associated with larger waist circumference (Odds Ratio 1.03 per 1.0 cm, *p* = 0.001), hypertension (Odds Ratio 2.7, *p* < 0.001), lower high-density lipoprotein cholesterol (Odds Ratio 0.3 per1.0 mmol/L, *p* = 0.002) and insomnia (Odds Ratio 2.0, *p* = 0.002). In adjusted analyses, prediabetes was associated with smoking (Odds Ratio 3.3, *p* = 0.001), hypertension (Odds Ratio 1.5, *p* = 0.03), and non-participation in cardiac rehabilitation (Odds Ratio 1.7, *p* = 0.003). In patients with type 2 diabetes, a higher HbA_1c_ level was associated with ethnic minority background (standardized beta [*β]* 0.19, *p* = 0.005) and low drug adherence (*β* 0.17, *p* = 0.01). In patients with prediabetes or normal blood glucose, a higher HbA_1c_ was associated with larger waist circumference (*β* 0.13, *p* < 0.001), smoking (*β* 0.18, *p* < 0.001), hypertension (*β* 0.08, *p* = 0.04), older age (*β* 0.16, *p* < 0.001), and non-participation in cardiac rehabilitation (*β* 0.11, *p* = 0.005).

**Conclusions:**

Along with obesity and hypertension, insomnia and low drug adherence were the major modifiable factors associated with type 2 diabetes, whereas smoking and non-participation in cardiac rehabilitation were the factors associated with prediabetes. Further research on the effect of individual tailoring, addressing the reported significant predictors of failure, is needed to improve glycaemic control.

**Trial registration:**

Retrospectively registered at ClinicalTrials.gov: NCT02309255, December 5th 2014.

## Background

The prevalence of type 2 diabetes (T2D) is rapidly increasing with the obesity epidemic [1]. Diabetes is associated with doubled long-term mortality risk after coronary heart disease (CHD) events [2] and increases the risk of both macro- (i.e. cardiovascular events) and micro- (i.e. retino-, nephro- and neuropathy) vascular complications linearly with increasing blood levels of glycated haemoglobin (HbA_1c_) [3, 4]. Intensive glycaemic control by multidrug treatment regimen, as performed in randomized controlled trials, reduces the risk of micro- and macro-vascular complications in T2D patients [5], but no improvement in cardiovascular or all-cause mortality has been demonstrated.

Attempts to delay the onset of T2D and prevent or delay microvascular and macrovascular complications drew attention to prediabetes, an intermediate form of dysglycaemia between normal blood glucose and overt diabetes [6]. In the most recent US guidelines, prediabetes is defined as HbA_1c_ between 5.7 and 6.4% (39–47 mmol/mol) [7]. Prediabetes may progress to T2D in up to 50% of cases within 5 years [8]. Also, patients with prediabetes are at increased risk of subsequent cardiovascular events after myocardial infarction [9]. Healthy lifestyle changes and treatment with antidiabetic drugs, as previously shown in clinical trials, may prevent or reduce the risk of T2D and its complications by 40–70% [10], emphasising the need for early disease detection and optimal management.

A complex array of demographic, psychosocial and behavioural factors influences T2D management [11–13], in addition to the causal risk factors increasing age, family history of diabetes, and obesity [7]. It remains to be studied carefully how these factors are associated with prediabetes and T2D, in particular within the CHD patient cohorts. The determinants of these diabetic manifestations may help to identify patients at risk, and to develop individualized interventions that may prevent prediabetes to progress to T2D and the subsequent cardiovascular events in both groups.

The NORwegian CORonary (NOR-COR) Prevention Study identifies sociodemographic, medical, and psychosocial factors associated with unfavourable risk factor control after CHD events in a cohort representing daily clinical practice (phase I). Moreover, the project aims to apply the factors of importance for risk factor control in developing tailored interventions (phase II) [14]. The present cross-sectional exploratory analysis sought to identify factors associated with glycaemic status and control among CHD patients. We hypothesise that potentially modifiable factors of importance for glycaemic status and control may be identified for those with prediabetes and T2D.

## Methods

### Design and population

The design, methods, and baseline patient characteristics of the NOR-COR Study have been described elsewhere [14]. In brief, 1789 consecutive patients undergoing a first or recurrent coronary event or treatment (i.e. acute myocardial infarction, coronary artery bypass graft operation, or percutaneous coronary intervention) were identified from the hospital medical records over the 3 years (2011–14) prior to study inclusion. All coronary angiograms and revascularization procedures were performed by experienced invasive cardiologists and thoracic surgeons at Oslo University Hospital. Reasons for exclusion of 423 patients were cognitive impairment (*n* = 28), psychosis (*n* = 18), drug abuse (*n* = 10), short life expectancy due to end-stage organ failure, malignant disease (*n* = 136), death following discharge from index hospitalization to invitation for the follow-up visit (*n* = 160), not being able to understand Norwegian (*n* = 44), or other reasons (*n* = 27). Of the remaining 1366 eligible patients, 1127 (83%) gave their informed consent to participate in a clinical visit and complete a comprehensive questionnaire [14]. Data on glycaemic status at follow-up were missing in 26 patients, while 18 patients with type 1 diabetes were excluded due to the different pathophysiology. Thus, a total of 1083 (96.1%) patients were included in the present study.

The study was conducted at two Norwegian hospitals (Drammen and Vestfold Hospital Trust) with a total catchment area of 380,000 inhabitants, corresponding to approximately 7% of the Norwegian population. The catchment areas have a blend of city and rural district population representative of Norwegian geography, economy, age distribution, morbidity, and mortality [15]. The level of education in this coronary population is in line with national data on cardiovascular disease patients [15].

### Outcome assessment

The primary outcome variables were glycaemic status (T2D, prediabetes or normal blood glucose) and glycaemic control (HbA_1c_) at follow-up. The diagnosis of T2D was defined as either i) treatment with antidiabetic drugs or a T2D diagnosis recovered from hospital records at the time of the index event or ii) the presence of HbA_1c_ ≥ 6.5% (≥48 mmol/mol) at the follow-up visit. Normal blood glucose was defined by HbA_1c_ < 5.7% (< 39 mmol/mol), and prediabetes by HbA_1c_ between 5.7 and 6.4% (39–47 mmol/mol) at follow-up, without T2D diagnosis at the index event [7]. HbA_1c_ was categorized based on a single blood test. Non-fasting venous whole blood sampled in an EDTA-tube was analysed on a clinical chemistry analyzer (Tosoh G8, Tosoh Medics Inc., San Francisco, CA, US) at Drammen Hospital to avoid inter-laboratory bias [14].

### Covariates (sociodemographic, medical, and psychosocial factors)

Demographic and medical factors registered from hospital medical records at the time of the index event:

- Demographic factors (age, sex, ethnic minority background defined as 1st and 2nd generation patients born in Asia, Africa or South America). Coronary history and treatment, cardiovascular medication, antidepressant medication, and somatic comorbidity summarized according to the Charlson comorbidity index.
- Participation in cardiac rehabilitation programs was confirmed by the hospital medical records and separate lists from the cardiac rehabilitation departments. In Drammen, cardiac rehabilitation includes a multidisciplinary one-day ‘heart school’ and exercise training twice per week for 6 weeks with start-up 2–6 weeks following the index event. Vestfold provides a comprehensive, multi-disciplinary program with individual and group approaches, motivational interviewing, education and exercise described previously [14]. Start-up is 2–3 weeks after the index event, followed by visits twice per week for 5 weeks, with exercise duration up to 6 months.

At follow-up 2–36 months after the index event, the following study factors were provided from:

- *Blood samples:* Total cholesterol, low density lipoprotein (LDL) cholesterol, high density lipoprotein (HDL) cholesterol, HbA_1c_, and C-reactive Protein (CRP).
- *Clinical examination*: systolic and diastolic blood pressure measured with standardized procedures using a validated digital sphygmomanometer (Welch Allyn WA Connex ProBP 3400). Weight (nearest 0.5 kg), height (nearest 0.5 cm), waist circumference (nearest 0.5 cm).
- The self-report questionnaires [14, 16]:
  - ◦ Socio-demographic factors: marital status and education (low education was defined by completion of primary and secondary school only).
  - ◦ Medical factors: smoking, physical activity, current treatment with cardiovascular drugs, drug related side-effects and adherence (Poor drug adherence was defined by a score of > 2 on the Morisky 8-item medication adherence questionnaire).
  - ◦ Psychosocial factors: quality of life (Short-Form 12), anxiety and depression (Hospital Anxiety and Depression Scale (HADS), Type D personality (DS-14), insomnia (Bergen Insomnia Scale), illness perception (Brief illness perception questionnaire), and perceived risk perception.

The sociodemographic variables, somatic comorbidity including coronary history and treatment are descriptive factors, while the remaining medical and psychosocial factors are regarded as potentially modifiable [14]. The NOR-COR Study explores a comprehensive set of study factors in relation to the following six well-established modifiable risk factors of CHD: smoking, body mass index ≥30 kg/m^2^, low or no physical activity (i.e. < 30 min moderate activity 2–3 times/week and never or < 1 time/week, respectively) [17], blood pressure ≥ 140/90 [80 in T2D] mmHg, LDL-cholesterol ≥1.8 mmol/L, and unfavourable glycaemic control (i.e.T2D and prediabetes). In the present study, addressing the risk factors T2D and prediabetes as primary independent variables, the remaining five risk factors are included as co-variates as indicated.

### Statistical analyses

The descriptive associations between covariates and glycaemic status are presented as frequencies (%) and mean ± standard deviation (SD), as appropriate. The χ^2^ test was used to compare proportions, and independent samples t-test to compare mean differences between groups. To identify the sub-set of predictors, in which all variables were statistically significant when multivariately controlled, a backwards stepwise elimination procedure was used to fit a multivariable logistic model, starting with the set of all socio-demographic, medical, and psychosocial factors with a *p*-value < 0.10 in bivariate analyses. Odds ratios (OR) and 95% confidence intervals (CI) for unfavourable glycaemic status by study factors were calculated. A similar backwards-stepwise elimination procedure was used to explore the linear association between HbA_1c_ and study factors in patients with T2D and prediabetes or neither, respectively. Beta (β) coefficients (standard error) for unfavourable glycaemic control by study factors were calculated. *P*-values < 0.05 were considered significant. Statistical interactions between study factors in ordinary least squares regressions, using HbA1c as dependent variable, were tested by entering two-way interaction terms in the equation, one at a time. Somatic co-morbidity and number of coronary events prior to the index event were not included in the regression models, as they could be direct effects rather than putative causes of T2D and unfavorable glycemic control. Statistical analyses were performed using SPSS version 21.

## Results

Baseline sociodemographic, medical, and psychosocial factors differed by glycaemic status are shown in Table 1 and Table 2*.* ST-elevation myocardial infarction, non- ST-elevation myocardial infarction and stable/unstable angina were the index coronary events in 30% (*n* = 324), 50% (*n* = 542) and 20% (*n* = 217) of the patients, respectively. At the index hospitalization 16 % (*n* = 175) had a diagnosis of T2D recorded in the hospital records, whereas information about prediabetes was not available. The prevalence of T2D was 22% (*n* = 243) at follow-up 2–36 (median 16) months after the coronary event compatible with a further 6% (*n* = 68) having new-onset T2D, defined by HbA_1c_ ≥ 6.5% (≥46 mmol/mol). Prediabetes (HbA1c 5.7–6.4% [39–47 mmol/mol]) was present in 44% (*n* = 484) at follow-up. Mean age at follow-up in patients with T2D and prediabetes was 62.3 (SD 9.1) years and 63.3 (SD 8.9) years, respectively, while 19% (*n* = 69) and 22% (*n* = 108) were women. Mean HbA_1c_ was 7.4% (57 mmol/mol) (SD 1.3) in subjects with T2D, and 55% (*n* = 134) did not reach the recommended treatment target of HbA_1c_ < 7.0% (< 53 mmol/mol), while 35% (*n* = 85) had HbA_1c_ > 8% (> 64 mmol/mol). Antidiabetic medication was prescribed to 70% (*n* = 122) of those with T2D at the index hospitalization, of whom 9% (*n* = 15) used combination therapy with insulin.

**Table 1:** Sociodemographic and medical factors in coronary patients with normal blood glucose, prediabetes and type 2 diabetes

	Normal blood glucose	Prediabetes	Type 2 diabetes	*P*-Value	
	(*n* = 356, 32.4%)	(*n* = 484, 44.1%)	(*n* = 243, 22.4%)	Normal blood glucose vs. prediabetes	Type 2 diabetes vs. prediabetes
*Socio-demographic factors*					
Age in years at index event, mean (SD)	59.4 (9.9)	63.3 (8.9)	62.3 (9.1)	***	N.S.
Number of months from index event to follow-up, mean (SD)	16.2 (10.3)	17.0 (10.5)	18.3 (10.5)	N.S.	N.S.
Female sex, n (%)	69 (19.4)	108 (22.3)	49 (20.2)	N.S.	N.S.
Ethnic minority, n (%)^a^	5 (1.4)	10 (2.1)	18 (7.4)	N.S.	***
Living alone, n (%)	57 (16.0)	84 (17.4)	50 (21.7)	N.S.	N.S.
Low education, n (%)^b^	221 (62.1)	346 (71.5)	181 (76.1)	**	N.S.
*Medical factors*					
Coronary index diagnosis, n (%)					
Non-ST elevation myocardial infarction	170 (47.8)	247 (51.0)	123 (40.1)	N.S.	*
ST-elevation myocardial infarction	111 (31.1)	148 (30.6)	58 (23.9)	N.S.	N.S.
Stable or unstable angina	75 (21.1)	89 (18.4)	62 (25.5)	N.S.	N.S.
> 1 coronary event prior to the index event, n (%)	91 (25.6)	119 (24.6)	113 (46.5)	N.S.	***
Participation in cardiac rehabilitation, n (%)	183 (51.4)	206 (42.6)	139 (57.2)	*	***
Charlson co-morbidity score, mean (SD)	3.6 (1.4)	4.1 (1.3)	4.8 (1.5)	***	***
Chronic kidney disease (eGFR < 60), n (%)	30 (8.4)	58 (12.0)	45 (20.3)	N.S.	*
Antidepressant medication at the time of the index event, n (%)	16 (4.5)	16 (3.3)	21 (8.6)	N.S.	**
Coronary risk factors and medication at interview					
Low density lipoprotein cholesterol mmol/L, mean (SD)	2.15 (0.73)	2.09 (0.80)	2.07 (0.84)	N.S.	N.S.
Low density lipoprotein cholesterol ≥1.8 mmol/L, n (%)	222 (62.4)	273 (56.4)	122 (51.0)	N.S.	N.S.
High density lipoprotein cholesterol mmol/L, mean (SD)	1.19 (0.34)	1.16 (0.32)	1.02 (0.30)	N.S.	***
Current smoking, n (%)	49 (13.8)	115 (23.8)	53 (22.7)	***	N.S.
C-reactive protein in mg/L, mean (SD)	2.06 (2.13)	2.37 (2.73)	3.22 (2.89)	N.S.	***
C-reactive protein ≥2 mg/L, n (%)	123 (35.5)	172 (36.6)	131 (56.5)	N.S.	***
Physical activity < 30 min of moderate activity 2–3 times a week, n (%)	158 (44.5)	227 (48.4)	129 (54.2)	N.S.	N.S.
Physical activity < 1 time a week, n (%)	53 (14.9)	73 (15.1)	59 (24.9)	N.S.	**
Blood pressure ≥ 140/90 (80 in diabetes) mmHg, n (%)	118 (33.1)	198 (44.0)	129 (60.6)	***	***
Waist circumference, mean (SD)	100.1 (11.1)	101.3 (12.1)	108.5 (12.6)	N.S.	***
Waist circumference ≥ 102/88 cm in men/women, n (%)	168 (51.5)	258 (57.5)	164 (76.6)	N.S.	***
Body Mass Index ≥30 kg/m^2^, n (%)	84 (23.6)	133 (27.5)	116 (54.2)	N.S.	***
Fruit and vegetables < 2 units/day, n (%)	132 (37.6)	187 (38.6)	92 (39.7)	N.S.	N.S.
Fish meals < 3 times/week, n (%)	171 (48.0)	213 (44.0)	118 (48.6)	N.S.	N.S.
Medication at interview, n (%)					
Statin, n (%)	332 (93.3)	451 (93.2)	224 (92.2)	N.S.	N.S.
High-intensity statin therapy, n (%)	168 (47.2)	247 (51.0)	104 (55.9)	N.S.	N.S.
Aspirin, n (%)	351 (98.6)	467 (96.5)	235 (96.7)	N.S.	N.S.
Beta-blockers, n (%)	248 (69.7)	349 (72.1)	188 (77.4)	N.S.	N.S.
ACE-inhibitors or ARB, n (%)	166 (46.6)	232 (47.9)	143 (58.8)	N.S.	**
Low Morisky Score^c^, n (%)	38 (10.7)	40 (8.3)	22 (9.6)	N.S.	N.S.

*SD* standard deviation, *NS* not significant, *eGFR* estimated glomerular filtration rate, *ACE* angiotensin converting enzyme, *ARB* angiotensin receptor blocker. *p < 0.05, **p < 0.01, ***p < 0.001

^a^Ethnic minority was defined as 1st and 2nd generation patients born in Asia, Africa and South America

^b^Low education was defined as completion of primary and secondary school only

^c^Scores: > 2 on the Morisky 8-item medication adherence questionnaire, indicating low adherence

**Table 2:** Psychosocial factors, quality of life, illness and risk factor perception in coronary patients with normal blood glucose, prediabetes and type 2 diabetes

Study factors	Normal blood glucose	Prediabetes	Type 2 diabetes	*P*-Value	
	(*n* = 356, 32.4%)	(*n* = 484,44.1)	(*n* = 243, 22.4%)	Prediabetes vs. normal blood glucose	Type 2 diabetes vs. prediabetes
*Psychosocial factors and quality of life*					
Hospital Anxiety and Depression Score-Anxiety ≥8, n (%)	66 (19.2)	99 (21.5)	53 (22.9)	NS	NS
Hospital Anxiety and Depression Score-Depression ≥8, n (%)	49 (14.0)	62 (13.3)	40 (17.2)	NS	NS
Type D personality disorder, n (%)	62 (17.4)	82 (16.9)	44 (18.7)	NS	NS
Worry score (Penn State Worry Questionnaire), mean (SD)	37.8 (12.4)	38.0 (13.0)	38.9 (12.5)	NS	NS
Insomnia, n (%)^a^	157 (44.1)	186 (38.4)	124 (54.1)	NS	***
Quality of life (SF-12), physical component summary, mean (SD)	39.0 (4.7)	38.3 (4.6)	37.9 (5.1)	*	NS
Quality of life (SF-12), mental component summary, mean (SD)	45.9 (6.1)	46.5 (6.4)	45.0 (6.8)	NS	**
*Illness and risk factor perception (1–10 Likert scale), mean (SD)*					
What do you feel is the likelihood of having a new heart attack over the next 12 months?	2.5 (2.3)	2.7 (2.4)	3.1 (2.6)	NS	NS
How much do you feel you can help reduce your risk of having another heart attack?	6.4 (2.7)	6.6 (2.8)	6.1 (2.8)	NS	*
How much do you think you will have to restrict your activities in the long-term due to your heart condition?	3.0 (2.7)	3.3 (2.7)	4.1 (2.9)	NS	***
*Brief Illness Perception (1–10 Likert scale), mean (SD)*					
How much does your illness affect your life? (consequences)	3.6 (2.8)	3.6 (2.8)	4.3 (2.9)	NS	**
How long do you think your illness will continue? (timeline)	7.7 (3.3)	7.6 (3.3)	7.9 (3.0)	NS	NS
How much control do you feel you have over your illness? (personal control)	5.9 (2.8)	5.9 (2.9)	5.9 (2.7)	NS	NS
How much do you think your treatment can help you? (treatment control)	7.3 (2.3)	7.4 (2.4)	7.2 (2.4)	NS	NS
How much do you experience symptoms from your illness? (identity)	3.0 (2.5)	3.0 (2.7)	4.0 (2.9)	NS	***
How concerned are you about your illness? (concern)	3.5 (2.8)	3.6 (3.0)	4.0 (3.1)	NS	NS
How well do you feel you understand your illness? (understanding)	6.9 (2.6)	7.0 (2.6)	6.7 (2.5)	NS	NS
How much does your illness affect you emotionally? (emotional response)	3.4 (3.0)	3.4 (3.0)	4.0 (3.0)	NS	*

*SD* standard deviation, *NS*; not significant, *SF* short form*p < 0.05, **p < 0.01, ***p < 0.001

^a^Insomnia (Bergen insomnia scale): a 7 -item self-report inventory designed to assess primary insomnia

Compared to patients with prediabetes, those with T2D were more frequently of ethnic minority background, had more somatic comorbidities, lower coronary risk factor control, and higher levels of CRP. They also had a higher cardiac rehabilitation participation rate, and reported insomnia more frequently. Compared to patients with normal glucose levels, those with prediabetes were significantly older, less educated, had more somatic co-morbidity, lower cardiac rehabilitation participation rate, higher prevalence of daily smoking, and more unfavourable blood pressure control.

A multi-adjusted logistic regression analysis showed the independent determinants of T2D compared to prediabetes, as well as of prediabetes compared to normal blood glucose (Table 3). Larger waist circumference, unfavourable blood pressure control, lower HDL cholesterol, insomnia, and perceived restriction in future activities due to the coronary disease were statistically significant study factors associated with T2D. The quality of life mental component was inversely related to T2D. Older age, non-participation in cardiac rehabilitation, current smoking, and unfavourable blood pressure control were statistically significantly associated with prediabetes.

**Table 3:** Multivariable^a^ odds ratios (confidence intervals and *p*-values) for prediabetes and type 2 diabetes by study factors

Study factors	Prediabetes (1) vs. normal blood glucose (0)		Type 2 diabetes (1) vs. prediabetes (0)	
	Odds ratio (95% CI)		Odds ratio (95% CI)	
Age at index event per 1.0 year	1.05 (1.03–1.07)	*<* 0.001		NS
Waist circumference per 1.0 cm		NS	1.03 (1.01–1.05)	0.001
Non-participation in cardiac rehabilitation	1.69 (1.19–2.40)	0.003		NS
Current smoking	3.29 (2.00–5.42)	0.001		NS
Blood pressure ≥ 140/90 (80 in diabetes) mmHg	1.48 (1.04–2.11)	0.030	2.67 (1.74–4.11)	0.001
Quality of life (SF-12), mental component per 1.0 points		NS	0.97 (0.94–0.99)	0.042
Insomnia^b^		NS	1.98 (1.30–3.05)	0.002
How much do you think you will have to restrict your activity in the long term due to your heart condition? per 1.0 points^c^		NS	1.14 (1.06–1.23)	0.001

*SD* standard deviation, *NS*: not significant, *CI* confidence interval

^a^Multi-adjusted models using backward step-wise elimination in binary logistic regression analyses adjusted for study and starting with risk factors showing *p* < 0.1 in bivariate association

^b^Insomnia (Bergen insomnia scale): a 7 -item self-report inventory designed to assess primary insomnia

^c^Measured by the Perceived risk perception Questionnaire (1–10 Likert scale)

A multi-adjusted linear regression analysis showed the independent determinants of HbA_1c_ in patients with T2D and in patients with either prediabetes or normal blood glucose level (Table 4). Ethnic minority background and low drug adherence were statistically significant study factors associated with higher HbA_1c_ levels in patients with T2D. Older age, higher waist circumference, current smoking, non-participation in cardiac rehabilitation and unfavourable blood pressure control were factors associated with higher HbA_1c_ in patients with prediabetes or normal blood glucose. The positive gradient between HbA1c and age was significantly stronger among those with prediabetes or normal blood glucose compared to those with T2D (F 32.4, *p* < 0.001). A corresponding stronger association between HbA1c and non-participation in cardiac rehabilitation (F 18.2, *p* < 0.001) and unfavourable blood pressure control (F 7.6, *p* = 0.006) was found. The positive association between HbA1c and ethnic minority background (F 17.2, *p* < 0.001) and low drug adherence (F 29.2, *p* < 0.001) was significantly stronger among those with T2D compared to those with prediabetes or optimal blood sugar.

**Table 4:** HbA1c regressed on study factors by multi-adjusted^a^ linear regression analysis

	Prediabetes or normal blood glucose (*n* = 840)			Type 2 diabetes (*n* = 243)		
Study factors	b (standard error)	Standardized *β*		b (standard error)	Standardized *β*	
Age at index event per year	0.006 (0.001)	0.157	*p* < 0.001			
Ethnic minority background			NS	1.06 (0.370)	0.193	*p* = 0.005
Non-participation in cardiac rehabilitation	0.074 (0.027)	0.106	*p* = 0.005			NS
Waist circumference per 1.0 cm	0.003 (0.001)	0.131	*p* < 0.001			NS
Current smoking	0.157 (0.033)	0.177	*p* < 0.001			NS
Blood pressure ≥ 140/90 (80 in diabetes) mmHg	0.056 (0.027)	0.079	*p* = 0.037			NS
Low self-report drug adherence^b^			NS	0.668 (0.265)	0.168	*p* = 0.012

*NS* not significant

Unstandardized (b) and standardized (*β)* regression coefficients. ^a^Adjusted for all variables with *p* ≤ 0.10 retained in backward elimination linear regression analysis

^b^Scores: > 2 on the Morisky 8-item medication adherence questionnaire indicating low adherence

## Discussion

In this cohort representing routine clinical practice, 2 of 3 patients had either prediabetes or T2D 2–36 months after hospitalization for a coronary event. The novelty of the present study is the detailed analysis of sociodemographic, medical and psychosocial factors providing new knowledge that potentially may improve risk factor control in the CHD population with disturbed glucose metabolism. The stratification of CHD patients into T2D, prediabetes and normal glucose control has been reported only once previously, in a US population without cardiovascular disease [18], and thus contributes to the originality of the present findings. Differences in the strength of associations of the potentially modifiable factors across the levels of glycemic status are demonstrated in the present study. For example, non-participation in cardiac rehabilitation emerges as a particular challenge in patients with prediabetes, while insomnia seems to be of particular importance for patients with T2D. The small sub-group of patients with ethnic minority background deserves particular attention due to their high risk of T2D and poor glycaemic control, as previously reported [19]. Patients with prediabetes at a cardiac event emerge as a particularly relevant subgroup to address more carefully in future secondary prevention of CHD.

The coronary index events in the present study are a ST-elevation myocardial infarction [20], and angina/non-ST-elevation myocardial infarction diagnoses [21]. Differences in pathophysiology, presentation and acute and long-term management of prediabetes and T2D could potentially influence our study results [20, 21]. Even though the relative number of patients with prediabetes and T2D varied by the coronary index diagnosis, the type of index event was not associated with glycemic status or control in adjusted analyses. An elegant randomised controlled study by Marfella R et al. demonstrated that optimal peri-procedural glycemic control up-regulated endothelial progenitor cell level and differentiation during acute ST-elevation myocardial infarction, with the results supporting the notion that tight glycemic control may improve myocardial salvage [22]. In our study, however, glycaemic status was assessed 2–36 months after the coronary event, and whether these mechanisms may explain any potential beneficial effect of tight glycemic control several months after the acute event is unknown.

Although long-term mortality after coronary events has declined in line with improved acute treatment and better risk factor management, patients with T2D are at particular risk for recurrent cardiovascular events [23]. Similarly, a meta-analysis has also demonstrated that compared to subjects with normal glycaemic status, those with prediabetes experienced a 20% increased risk for cardiovascular disease [9]. The mechanisms behind the poor prognosis in patients with T2D and prediabetes are not completely understood, but a higher prevalence of complications in combination with lack of appropriate secondary preventive treatment contribute [24]. It was therefore not unexpected that patients with T2D had most somatic comorbidty and poorest overall risk factor control, followed by subjects with prediabetes. Subjects with normal blood glucose had least comorbidity and best coronary risk profile. Interestingly, T2D patients were characterized by central obesity, unfavourable blood pressure, and low levels of HDL cholesterol, which constitute important components of the metabolic syndrome [25].

The T2D patients in our study were characterized by subclinical inflammation. The association between high-sensitivity CRP and T2D, however, was no longer significant when adjusting for somatic comorbidity and other cardiovascular risk factors in our study. Inflammation, particularly in diabetes, plays a pivotal role in the series of events that result in plaque disruption and thus influence the incidence and severity of cardiovascular events in T2D patients [26]. The involvement of the gene regulating transcription factor sirtuin 6 in the inflammatory diabetic atherosclerotic lesions was recently established. Incretin-based therapies (i.e. GLP-1 receptor agonists, and dipeptidyl peptidase-4 inhibitors) resulted in a greater sirtuin 6 expression and less inflammation and oxidative stress in carotid plaques of diabetic versus non-diabetic patients, indicating a more stable plaque phenotype [27]. The prevalence and predictors of culprit coronary plaque rupture identified by optical coherence tomography in patients undergoing coronary angiography was recently described in a meta-analysis by Iannaccone et al. which showed high rates of plaque rupture in patients with acute coronary syndrome [28]. Hypertension was the only clinical predictor for ST-elevation myocardial infarction, whereas increasing age, diabetes and hyperlipidemia were clinical predictors in non- ST-elevation myocardial infarction and unstable angina [28]. Another interesting endocrinological aspect of inflammation is the association with subclinical hypothyroidism independent of the traditional risk factors, as demonstrated by Marfella et al. [29]. Their study showed a potential interplay between subclinical hypothyroidism and inflammatory activity in atherosclerotic plaque progression towards instability, and that synthetic levothyroxine replacement therapy might contribute to plaque stabilization and thus improved prognosis by inhibiting the immunity-dependent plaque rupture in patients with subclinical hypothyroidism [29].

Low self-reported drug adherence was significantly associated with higher HbA_1c_ level in T2D, but not in prediabetic patients. Among patients with T2D having HbA_1c_ > 8.0% (64 mmol/mol), 30% did not use antidiabetic drugs, while only 9% used combination therapy with antidiabetic drugs and insulin. Intensified antidiabetic treatment and strategies to improve drug adherence therefore appear to be the major factors needed to improve glycaemic control in T2D patients. The newer hypoglycemic drugs, in particular incretins, are recommended for T2D patients with established CHD [6], and their potential beneficial pleiotropic effects on atherosclerotic plaque functionality and thus clinical outcome was recently documented in patients with a non-obstructive coronary artery stenosis non-ST-elevation myocardial infarction event [30].

In a long-term follow-up of patients with T2D and microalbuminuria without CHD participating in the Steno-2 trial, Gæde et al. showed that intensive target-driven intervention with multiple drug combinations and behaviour modification had sustained beneficial effects on the incidence of cardiovascular events and mortality compared to conventional multifactorial treatment [31]. The intensive drug regimen included higher dosages of statins and ACE-inhibitors than that of conventional treatment, resulting in lower LDL cholesterol and systolic blood pressure. These findings emphasize the importance of adequate prescription of and adherence to secondary preventive drug therapy in T2D patients.

The risk factors obesity, smoking and unfavorable blood pressure control were also significantly associated with increasing HbA_1c_ level in the group of patients with prediabetes or normal blood glucose, but not in those with T2D. The pharmacological treatment of T2D, aiming at HbA_1c_ control, may partly account for these differences at group level. The present finding thus emphasizes the particular need to address glycemic control through appropriate lifestyle changes and drug treatement, even in CHD patients without T2D [10].

Participation in multidisciplinary cardiac rehabilitation programme is strongly recommended and has proven beneficial for several cardiovascular risk factors in CHD patients with and without diabetes [32]. Participation in a comprehensive cardiac rehabilitation program was associated with improved risk profile as well as drug adherence in a recent publication derived from our data set [33]. In the present study, the participation rate in cardiac rehabilitation was lowest in patients with prediabetes, and was significantly related to increasing HbA_1c_ level. Even though patients with T2D had the highest participation rate in cardiac rehabilitation, more than 40% did not participate. Efforts to increase the participation rate in effective preventive cardiac rehabilitation programs are of particular importance for both patients with prediabetes and T2D, as non-participation appears to contribute to poor risk factor control.

No differences in psychosocial factors (i.e. anxiety, depression, worry, type D personality illness perception) were found in patients with prediabetes or with T2D in the present study, apart from the higher prevalence of insomnia among T2D compared with prediabetic patients. Psychosocial factors like diabetes distress, depression, and anxiety are prevalent in general populations with diabetes [34], and associated with increased risk of developing T2D in subjects with prediabetes [35]. The present selection of CHD patients may in part account for differences when compared with studies of diabetic populations. Furthermore, the level of psychosocial distress may decrease with increasing time since CHD events [36] and the association between psychosocial factors and glycaemic status may potentially be different if these factors had been assessed at the time of the index event. According to a recent study [12], depression and other psychosocial factors were not associated with the HbA_1c_ level in patients with T2D. Diabetes distress has recently been suggested to be more important for glycaemic control in patients with diabetes than clinical depression or depressive symptoms [13, 37].

The prevalence of insomnia accords to previous reports from the general population [38] and T2D patients [39]. A significant relationship between sleep problems and increased HbA_1c_ has been found in most, but not all, studies [40]. Patients with T2D and sleep problems have been considered a high-risk group for disease progression and might have resistance to standard treatment for T2D [40]. The pathways linking insomnia and glycaemic control remain unknown and are not determined in our study applying a cross-sectional design. The high prevalence of insomnia in T2D in the present study favours the previous recommendation that screening for sleep disturbance should be part of the management of T2D [41]. Sleep disorders are in general likely to be under-recognized and under-reported in the health care system [38]. Effective interventions to improve insomnia (pharmacotherapy and cognitive behaviour therapy) are available [40]. Interestingly, few studies have addressed the relationship between sleep and prediabetes. We did not find a significant association between insomnia and prediabetes, as reported by two previous studies excluding patients with CVD [42, 43]. Selection bias and differences in assessment methods may explain the differences. Separate insomnia symptoms (e.g. sleep onset, maintenance, early awakening, feeling adequately rested) were not explored separately in the present study. Which insomnia/sleep symptoms may have the strongest associations with prediabetes and T2D should be explored in future studies.

Immigrants from Asia and Africa living in Europe have a higher prevalence of T2D and are diagnosed earlier in life compared to native Europeans [44, 45]. In line with this, our coronary population had a three times higher prevalence of first and second generation ethnic minorities among patients with T2D compared to those with normoglycemia or prediabetes. Similar to several earlier studies [19], our study also found ethnic minorities with T2D to have inferior glycaemic control. Ethnic disparities in sociodemographic, cultural, and psychosocial factors may contribute to the observed ethnic inequalities in diabetes management [46]. In this respect, the high frequency of clinically significant symptoms of anxiety (29%), depression (24%), and insomnia (75%) observed in the subgroup with ethnic minority background, is a potentially important observation in the present study. Although these results must be interpreted with care due to the small numbers, the findings call for further studies to explore the role of psychosocial factors in ethnic minorities. Furthermore, these findings may encourage clinicians to recognize psychosocial distress as a potential barrier to secondary prevention in cardiovascular disease and in the management of T2D.

The proportion of patients with known T2D at the time of the index event (16%) was lower than reported in the latest EuroAspire survey (33%) [47], with comparable inclusion criteria including age distribution, and prevalence of obesity. Patient inclusion from largely academic centres [47] with potentially more focus on coronary prevention as well as more systematically screening for T2D than in everyday clinical practice, may, in part, account for the difference. At follow-up, an additional 6% of our patients fulfilled the diagnostic criteria for T2D, whereas the prevalence of undetected T2D in EuroAspire was 19%. Differences in prevalence rates may be explained by differences in measurements, as the latter study also screened the patients by an oral glucose tolerance test [47]. Systematic screening with both HbA_1c_ and oral glucose tolerance test has recently been proven to identify more individuals with T2D than each of them alone [48]. Thus, more patients with T2D would potentially have been diagnosed in the present study if screened by both tests. This highlights the need for better integration of systematic screening strategies for T2D after hospitalization for coronary events.

### Study limitations and strengths

Most study factors in the present study were measured at one point in time and are thus prone to measurement and recall bias. However, a reproducibility study of the NOR-COR questionnaire demonstrated highly acceptable test-retest values for all key items and instruments [16]. The sample size does not allow sub-group analyses (e.g. age and gender) in the subgroup with T2D. The validity of using a single HbA1c measurement for the diagnosis of diabetes is suboptimal, as WHO guidelines advocate at least one additional HbA1c or plasma glucose test result with a value in the diabetic range in order to confirm the diagnosis; either fasting, from a random (casual) sample, or from the oral glucose tolerance test (OGTT) [49]. For the index hospitalization, however, diabetic status was based upon treatment with antidiabetic drugs or a T2D diagnosis recovered from hospital records at the time of the index event. Since diabetes diagnostics were not performed routinely at the time of the index event, the proportion of patients receiving their first T2D diagnosis during the index hospitalization remains unknown. Due to this limitation of the diagnostic test, some asymptomatic patients with diabetes may have been missed at follow-up. Our diagnostic criteria are, however, in accordance with the most recent US guidelines [7] and those of the WHO [49]. Furthermore, reporting bias of the T2D diagnosis in the hospital medical records might have occurred. Accordingly, some of the patients classified as new-onset T2D based HbA_1c_ at follow-up may have also had known T2D at the time of the index event. Our study design did not incorporate echocardiographic measurements in order to detect possible deterioration in left ventricular function in patients with ST-elevation myocardial infarction who had conventional glycaemic control. Although this study provides a comprehensive evaluation of determinants associated with prediabetes and T2D, additional confounders should be considered, including family history of diabetes, the duration of T2D and details on T2D management in general practice. Moreover, information about the different oral antidiabetic drugs prescribed at the time of the index coronary event and the reasons for admission to or non-participation with cardiac rehabilitation were not available. We did not assess sleep duration and objective measures of sleep and did not control for use of hypnotics, sedative, use of caffeine and obstructive sleep apnoea syndrome. The retrospective design with follow up times of 2–36 months after the index coronary event gives some indication of how HbA_1c_ and the study factors vary by time. A prospective design with repeated measurements is required to explore the influence of time properly. The representative cohort from routine clinical practice, the high participation rate (i.e. 83%) and the comprehensive data set are important strengths of the study.

## Conclusion

Prediabetes and T2D are prevalent conditions both prior and subsequent to coronary events. Along with obesity and hypertension, insomnia and low drug adherence were the major modifiable factors associated with T2D, whereas smoking and non-participation in cardiac rehabilitation were the factors associated with prediabetes. Further research on the effect of individual tailoring, addressing the reported significant predictors of failure, is needed to improve glycaemic control.

## Abbreviations

CHD: Coronary heart disease
CRP: C-reactive protein
HbA_1c_: Glycated haemoglobin
HDL-C: High-density lipoprotein-cholesterol
LDL-C: low-density lipoprotein-cholesterol
T2D: Type 2 diabetes
